# The efficacy of pegylated interferon alpha-2a and entecavir in HBeAg-positive children and adolescents with chronic hepatitis B

**DOI:** 10.1186/s12887-022-03482-0

**Published:** 2022-07-20

**Authors:** Yi He, Yingzhi Zhou, Huimin Wang, Xiaorong Peng, Yunan Chang, Peng Hu, Hong Ren, Hongmei Xu

**Affiliations:** 1 Department of Infection, Children’s Hospital of Chongqing Medical University, National Clinical Research Center for Child Health and Disorders, Ministry of Education Key Laboratory of Child Development and Disorders. Chongqing Key Laboratory of Child Infection and Immunity, Chongqing Medical University, 136, Zhongshan Road, Yuzhong District, Chongqing, 400014 People’s Republic of China; 2 Department of Infectious Diseases, Key Laboratory of Molecular Biology for Infectious Diseases (Ministry of Education), Institute for Viral Hepatitis, The Second Affiliated Hospital, Chongqing Medical University, Chongqing, People’s Republic of China

**Keywords:** Chronic hepatitis B, Paediatrics, Entecavir, Pegylated interferon, Treatment

## Abstract

**Background and objectives:**

Pegylated interferon alpha-2a (peg-IFN α-2a) and entecavir (ETV) are both recommended as the first-line antiviral drugs for chronic hepatitis B (CHB) at present. We aimed to compare the efficacy and safety between peg-IFN α-2a and ETV initial therapy in children and adolescents with CHB and investigate the potential factors affecting the treatment response during the first 48 weeks.

**Methods:**

We retrospectively selected 70 treatment-naïve children and adolescents with CHB who received peg-IFN α-2a(*n* = 26) or ETV(*n* = 44) as initial therapy and completed 48-week follow-up for data analysis. Blood samples before treatment were collected from 26 patients of the cohort to assess the cytokine profiles.

**Results:**

We found that initial peg-IFN therapy results in higher rates of hepatitis B surface antigen (HBsAg) serological response (SR) but lower rates of virological and biochemical response rates compared to ETV at week 48. As for achieving hepatitis B e antigen (HBeAg) SR, peg-IFN was comparable to ETV in the univariate analysis and turned out to be better than ETV after adjustment for important baseline factors. We also found that elevated pre-treatment IL-18 level was significantly associated with HBeAg SR, and remained as the only independent factor of predicting HBeAg SR after adjustment for other important factors. No serious adverse effects of the 2 drugs were reported during the 48-week follow-up.

**Conclusions:**

comparing to ETV, peg-IFN was superior in achieving HBsAg and HBeAg SR; higher baseline IL-18 levels were independently associated with HBeAg SR in this study of children and adolescents with CHB.

**Supplementary Information:**

The online version contains supplementary material available at 10.1186/s12887-022-03482-0.

## Introduction

Despite the availability of infant vaccination programs and potent antiviral therapies, the prevalent infection of hepatitis B virus (HBV) has long been a health burden. Compared to the infection acquired in adulthood, HBV transmission via the mother-to-infant route or in early childhood leads to a higher risk of immune tolerance and chronicity of HBV [1]. Theoretically, early intervention of chronic hepatitis B (CHB) can prevent disease progression to liver cirrhosis, hepatocellular carcinoma, liver failure or other end-stage events. However, due to the low coverage of therapies and the limited reference data for the treatment of children with CHB, the effectiveness of antiviral regimens has not yet been fully proven in paediatric patients [2].

The variety of drugs adds complexity to the choice of therapy regimen. With the development of antiviral therapies over the past few decades, interferon alpha (IFNα) or pegylated interferon (peg-IFN) and high genetic barrier nucleos(t) ide analogues (NAs) such as entecavir (ETV), are currently recommended as first-line therapies in children with CHB [3, 4]. The approved drugs for paediatric CHB patients are limited because of the strict licenced ages and insufficient clinical data on efficacy and safety. ETV is recommended for patients ≥2 years old and IFNα is recommended for patients ≥1 year old under close monitoring. With the approval of peg-IFN α-2a for CHB paediatric patients(≥3 years old) by the United States of America and the European Union in 2017, peg-IFN α-2a has also recently been recommended in paediatric CHB patients by international guidelines. Due to the less frequent injection and prolonged stability in vivo of peg-IFN compared to IFN-ɑ, peg-IFN is promising to replace the use of IFN-ɑ [5]. ETV and peg-IFN, both of which are first-line drugs and are the most commonly used in paediatric patients, have distinctly different mechanisms in controlling the progression of CHB. ETV, one of the NAs competitively inhibiting HBV polymerases, is highly potent in viral suppression and is generally well-tolerated, but has resistance limitations and requires a prolonged treatment duration. Peg-IFN, an immunomodulatory agent, has a finite treatment duration, no resistance, and achieves higher hepatitis B e antigen (HBeAg) and hepatitis B surface antigen (HBsAg) serological response (SR) rates than NAs, but is not extensively used due to high costs and side effects [6, 7]. A previous study compared the treatment response of continuous ETV monotherapy and finite peg-IFN α-2b initial therapy in CHB adults after a median of 92-week follow-up, and found that peg-IFN had relatively higher rates of HBeAg and HBsAg SR, while ETV had relatively higher rates of HBV DNA undetectability [8]. The 2 drugs have unique non-overlapping advantages, and the optimal choice of initial treatment between ETV and peg-IFN for individual patients remains controversial, especially for children and adolescents with CHB, considering the limited clinical data in this population. In addition, the immune status and tolerability of drugs between paediatric and adult patients might be different; thus, referring to studies of adults might be inappropriate. Taking these controversies into consideration, more clinical data regarding the efficacy and safety of these 2 drugs for CHB children and adolescents are required. In this retrospective observational study, we comprehensively evaluated the treatment response and adverse effects in treatment- naïve CHB children and adolescents receiving peg-IFN α-2a or ETV as an initial antiviral therapeutic regimen in a 48-week follow-up.

Host immune factors, especially cytokine balance, play a key role in the immune response against pathogens and influence the disease outcome of HBV infection (clinical recovery or persistent infection). Previous studies have revealed a significant association between host immune cytokine profiles and disease progression or treatment response [9, 10]. Cytokines can act not only as signalling molecules that initiate and regulate downstream immune activities by binding to the specific receptors of target cells, such as NK cells and cytotoxic T cells, but also, as effector molecules that directly affect viral replication [11]. For example, IFN can promote antigen presentation to CD8+ T cells as well as phagocytosis in NK cells and monocyte–macrophages. It has been reported that the induction of serum IFN-γ levels during treatment is associated with virological control and HBeAg seroconversion [12]. Cytokines play various roles in HBV infection, including cellular immune responses [e.g.,interleukin (IL)-2, interferon (IFN)-γ], humoral immune responses (e.g., IL-4, IL-6, IL-10), immunomodulation and immunosuppression [e.g., tumor growth factor-beta (TGF-β), IL-10], and mediating inflammation(e.g., IL-17, IL-22,IL-23) [13]. To identify the potential immune signatures of responders to treatment, a series of cytokines related to CHB were chosen after a literature research, and an investigation of pretreatment cytokine profiles was performed in the study population.

## Methods

### Study population

CHB children and adolescents aged 2–18 years who attended the Children’s Hospital of Chongqing Medical University and started antiviral treatment from Jan 2013 to Oct 2020 were systematically reviewed with electronic medical records and laboratory inspection. Currently, ETV and peg-IFNα-2a are both first-line drugs for CHB. For patients up to the licenced age of the 2 drugs, the antiviral therapy (ETV or peg-IFN) was selected according to the patients and their parents’ choice. Doctors informed the patients and their parents of the route of administration of these 2 drugs, the duration of treatment, related side effects, indications and contraindications before selecting the antiviral therapy. Eligible patients were HBeAg-positive with elevated alanine aminotransferase (ALT) (> 1.5 times the upper limit of normal) and treatment-naïve at baseline, who received Peg-IFN α-2a (Pegasys, Roche) or ETV (Entecavir Dispersible Tablets, Chia Tai Tianqing) and completed the regular follow-ups for at least 48 weeks. The exclusion criteria were as follows: patients who received antiviral treatments other than Peg-IFN or ETV; patients with hepatic decompensation, hepatocellular carcinoma, liver failure, liver transplantation, chronic immunosuppression, coexistence of other liver diseases (e.g. autoimmune hepatitis, drug-induced liver injury or Wilson’s disease) or coinfection of other hepatotropic virus (hepatitis A, C, D, E, or human immunodeficiency virus); patients who did not completed the 48-week treatment or lost follow-up. In this retrospective observational study, a total of 70 CHB children and adolescents who met the inclusion criteria were included in the data analysis, consisting of 44 patients receiving ETV mono-therapy(0.03 mg/(kg*day)) and 26 patients initiating with Peg-IFN α-2a mono-therapy(104μg/(m^2^*week)). The study was conducted according to the guidelines of the Declaration of Helsinki and was approved by the Ethics Committee of the Children’s Hospital of Chongqing Medical University (No:2019 []), and written informed consents were obtained from the children’s guardians.

### Clinical parameter assessment

Patient case report forms were used to abstract medical records for demographic and clinical information. Information on their diagnosis and treatments was obtained by review of laboratory, pathological, radiological, and clinical records. Biochemical tests were performed using routine automated analyzers. Serum HBV DNA levels were measured by real time fluorescence quantitative polymerase chain reaction assays (Sansure Biotech,China), and the lowest limit of detection was 400 IU/mL. Serum HBsAg and HBeAg level were detected using commercial Chemiluminescence Microparticle Immuno Assay (CMIA) kits (Abbott GmbH & Co. KG, Wiesbaden, Germany). The lower limit of quantitative HBsAg determination was 0.05 IU/ml, and HBeAg-positive at values ≥1 S/CO (sample rate/cut off rate). The HBV genotypes were measured by real-time fluorescence quantitative polymerase chain reaction assays (Daan, China). HBV genotypes were determined by direct sequencing of the pre-S/S gene and comparison with the reference sequences in GenBank (NCBI). Grade of Inflammation and stage of fibrosis in liver histology were evaluated according to Scheuer’s criteria on a scale of 0–4.

### Definition of outcomes

The evaluation of outcome was assessed at 48 weeks of antiviral therapy. The definition of outcomes was set according the international guideline for pediatric CHB [3]. HBeAg /HBsAg SR was defined as loss of HBeAg/HBsAg with or without the emergence of hepatitis B e antibody (HBeAb)/hepatitis B surface antibody (HBsAb). HBeAg seroconversion was defined as loss of HBeAg/HBsAg with the emergence of HBeAb/HBsAb. Biochemical response was defined as normalization of elevated ALT levels. Virological response was defined as HBV DNA < 2000 IU/ml, and an undetectable HBV DNA level in serum was < 400 IU/ml. Primary non-response was defined as less than 2 log_10_IU/ml decrease in HBV DNA levels from baseline after 24 weeks of therapy. Early virological response was defined as suppression of HBV DNA to undetectable (< 400 IU/mL) after 24 weeks of therapy. The safety of treatment was assessed by recording the adverse events at each visit as well as periodically monitoring the cardiogram, levels of blood ammonia and lactate for ETV group, and white blood cell counts, thyroid function and levels of autoantibodies for peg-IFN group. Virologic breakthrough was defined as more than 1 log_10_IU/ml increase of HBV DNA levels during therapy.

### Multiplex cytokine assay by quantibody® array kit

Serum samples were obtained from 26 of the included patients at baseline (on the day of therapy initiation) and stored at − 80 °C. A human chemokine quantibody® array kit (Raybiotech)-- a multiplexed sandwich ELISA-based quantitative array platform, was used per the manufacturer’s instructions. InnoScan 300 Microarray Scanner (Innopsys, arc d’Activités Activestre, 31,390 Carbonne-France) was used for fluorescence detection. The quantibody® array quantitatively measured the expression of a series of 51 chemokines (the list of cytokines was in Table S1). After reviewing the medical records and clinical data, we divided the 26 patients into 2 outcome subgroups depending on whether they achieved HBeAg SR (*n* = 13) or not (*n* = 13) after 48 weeks of treatment.

### Statistical analysis

Clinical data were analyzed with SPSS 26.0 (SPSS, Inc. Chicago, IL, United States). Normality test was applied to detect the distribution of data before further analyzing. Comparisons were performed using the Student’s t test or Mann-Whitney u test for continuous variables and the Chi-square test or the Fisher’s exact test for categorical variables, as appropriate. The association between HBeAg SR and baseline factors was assessed using univariate analysis and the binary logistic regression analysis. The classification of HBeAg SR and non-SR (NSR) using cytokine profiles was performed by the “Random Forest” package with R software (R Foundation for Statistical Computing, version 4.0.2). A fivefold cross-validation of random forest model was conducted to rank the contribution of each cytokine to discriminate HBeAg SR and NSR. Multidimensional scaling plot was used for visual comparison of cytokine levels between the 2 groups. The importance of individual cytokines was measured by Gini scores during the classification; cytokines with higher mean decreased Gini scores were considered to be more important than cytokines with lower numbers. Receiver operating characteristics (ROC) analysis was performed to evaluate the prediction value in HBeAg SR. All tests were 2-tailed, and *P* < 0.05 was considered as statistically significant.

## Results

### Characteristics of the study population

**Fig. 1:**
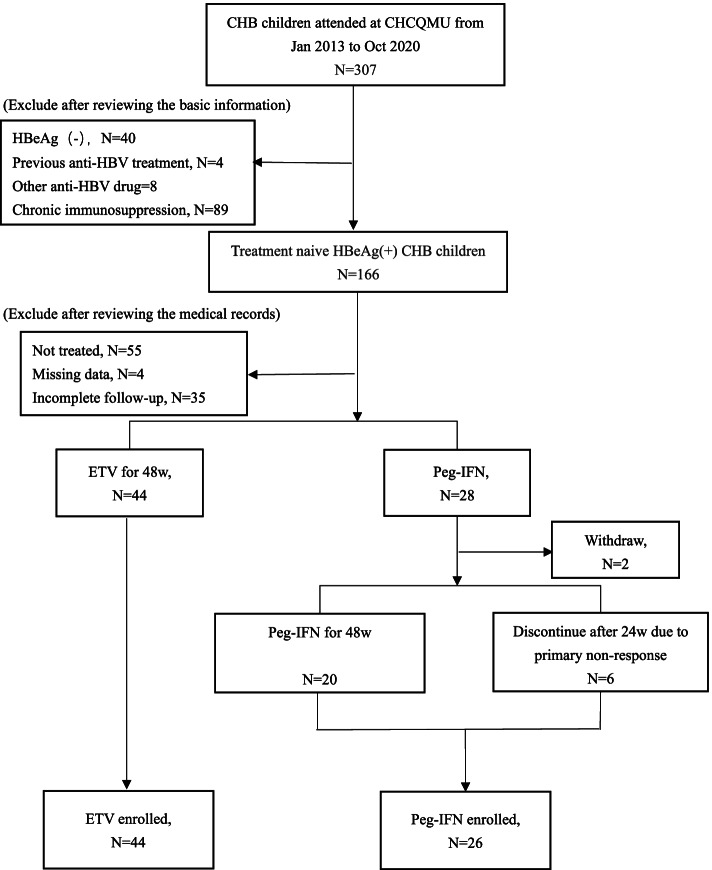
Selection process of the study population. CHB: chronic hepatitis B; IA: immune active; ETV: entecavir; peg-IFN: pegylated interferon; CHCQMU: Children’s Hospital of Chongqing Medical University

Overall, 70 CHB children and adolescents who met the inclusion criteria were included in the study, among whom 44 were initially administered ETV and 28 were initially administered peg-IFN. The selection process of the study population is shown in Fig. 1. No withdrawal occurred among the 44 patients in the ETV group. Among the 28 patients in the peg-IFN group, 2 withdrew before week 24 due to economic considerations and were not included in the data analysis. Six discontinued the drug at week 24 because the patients achieved a less than 2 log_10_ IU/ml reduction in serum HBV DNA and then went on the clinical follow-up to week 48. The demographic and clinical data of the study population at baseline are summarized in Table 1. There were no significant differences in age, weight, viral load or ALT levels; or in the distribution of sex, HBV genotype, grade of inflammation and stage of fibrosis in the liver pathology evaluation between the 2 treatment groups at baseline.

**Table 1:** Baseline characteristics of patients enrolled in the study

Parameters	Peg-IFN(*n* = 26)	ETV(*n* = 44)	t/x^2^	P
Male, N(%)	16(61.5%)	27(61.4%)	0	0.988
Age (year)	8.01(3.97)	6.92(4.00)	1.098	0.276
Weight (kg)	30.02(16.06)	25.24(13.33)	1.343	0.184
ALT/ULN	3.38(2.24)	4.95(3.97)	−1.851	0.069
AST/ULN	2.35(1.60)	3.26(2.65)	−1.594	0.116
Log_10_HBV DNA (IU/ml)	7.38(1.15)	7.46(1.03)	−0.306	0.761
HBsAg quantification (IU/ml)	38,575.9(51,834.5)	28,540.0(28,999.9)	0.935	0.354
HBeAg(s/co)	844.05(530.96)	910.67(497.47)	−0.528	0.599
HBV genotype (getotype B/genotype C/Undetected), N	11/6/9	21/13/10	1.208	0.547
Grade of inflammation in liver histology(G0–1/G2–3), N	10/16	22/22	0.877	0.349
Stage of fibrosis in liver histology(S0–1/S2–3), N	16/10	30/14	0.320	0.572
Treatment switch due to suboptimal HBV DNA reduce, N(%)	6(23.08%)	0(0%)	8.356	**0.002**

Continuous data were expressed as mean (standard deviation). *ETV* Entecavir, *Peg-IFN* Pegylated interferon, *ALT* Alanine aminotransferase, *AST* Aspartate aminotransferase, *ULN* Upper limit of normal, *HBV* Hepatitis B virus, *HBsAg* Hepatitis B surface antigen, *HBeAg* Hepatitis B envelop antigen

### Assessment of treatment efficacy

The median levels of HBV DNA, HBeAg and ALT at each visit (week 4, 12, 24 and 48) in the 2 treatment groups are shown in Table S2. The rates of HBeAg SR, HBsAg SR, virological response and biochemical response during the follow-up tended to increase in both groups, as shown in Fig. S1. The HBeAg SR rates at 4, 12, 24 and 48 weeks of treatment in the peg-IFN group were 0, 15.38, 23.08 and 46.15%, while the corresponding rates were 2.27, 11.36, 20.45 and 34.09% in the ETV group. The HBsAg SR rates at 24 and 48 weeks of treatment in the peg-IFN group reached 3.85 and 23.08%, respectively, while the corresponding rates were 0 and 4.54% in the ETV group. A virological response was achieved in 3.85, 15.38, 30.77 and 65.38% of patients treated with peg-IFN and in 15.91, 50.00, 84.09 and 86.36% of patients treated with ETV, respectively. The biological response was achieved in 7.69, 15.38, 26.92 and 46.15% of patients treated with peg-IFN and in 25.00, 43.18, 65.91 and 86.36% of patients treated with ETV, respectively.

The comparison of treatment efficacy between the 2 groups at week 24 and 48 is shown in Table 2. For SR, peg-IFN showed comparable effectiveness for HBeAg SR and better effectiveness for HBsAg SR compared to ETV. At these 2 time points, peg-IFN and ETV showed similar effectiveness in achieving HBeAg SR (26.92% vs. 20.45% at week 24, 46.15% vs. 34.09% at week 48, *P* > 0.05). Although there was no significant difference in achieving HBsAg SR between the 2 groups at week 24, the peg-IFN group achieved a higher rate of HBeAg SR at week 48(3.85% vs. 0% at week 24, *P* > 0.05; 23.08% vs. 4.54% at week 48, *P* < 0.05). For virological and biological responses, ETV showed better performance during the course than peg-IFN. At week 24 and week 48, the ETV group achieved higher rates of virological response than the peg-IFN group (54.55% vs. 19.23% at week 24, 68.18% vs. 53.85% at week 48, *P* < 0.05). In addition, at week 24, the ETV group achieved a lower rate of primary nonresponse and a higher rate of early virological response than the peg-IFN group (0% vs. 42.31 and 54.55% vs. 20.83% respectively, *P* < 0.05). The ETV group also achieved a higher rate of biological response than the peg-IFN group (65.91%% vs. 26.92%% at week 24, 86.36% vs. 46.15% at week 48, *P* < 0.05).

**Table 2:** Treatment effectiveness at week 24 and 48 of treatment

Time	Parameters	Peg-IFN (*n* = 26)	ETV (*n* = 44)	x^2^	P
Week 24	HBV DNA undetectable, n(%)	5(19.23%)	24(54.55%)	8.399	**0.004**
	HBV DNA < 2000, n(%)	8(30.77%)	37(84.09%)	20.239	**0.000**
	HBeAg serological response, n(%)	7(26.92%)	9(20.45%)	0.388	0.533
	HBeAg seroconversion, n(%)	6(23.08%)	9(20.45%)	0.067	0.796
	ALT normalization, n(%)	7(26.92%)	29(65.91%)	9.944	**0.002**
	HBsAg serological response, n(%)	1(3.85%)	0	1.717	0.371*
	HBsAg seroconversion, n(%)	1(3.85%)	0	1.717	0.371*
	Primary non-response	11(42.31%)	0	19.007	0.000*
	Early virological response	5(20.83%)	24(54.55%)	8.399	**0.004**
Week 48	HBV DNA undetectable, n(%)	14(53.85%)	30(68.18%)	1.439	0.230
	HBV DNA < 2000, n(%)	17(65.38%)	38(86.36%)	4.272	**0.039**
	HBeAg serological response, n(%)	12(46.15%)	15(34.09%)	1.004	0.316
	HBeAg seroconversion, n(%)	10(38.46%)	15(34.09%)	0.136	0.798
	ALT normalization, n(%)	12(46.15%)	38(86.36%)	12.948	**0.000**
	HBsAg serological response, n(%)	6(23.08%)	2(4.54%)	3.865	0.045*
	HBsAg seroconversion, n(%)	4(15.38%)	2(4.55%)	1.262	0.186*

*ETV* Entecavir, *PegIFN* Pegylated interferon, *ALT* Alanine aminotransferase, *AST* Aspartate aminotransferase, *ULN* Upper limit of normal, *HBV* Hepatitis B virus, *HBsAg* Hepatitis B surface antigen, *HBeAg* Hepatitis B envelop antigen

### Analysis of independent factors for the treatment response

Baseline variables including drugs (IFN vs. ETV), sex (male vs. female), age(< 6 years vs. ≥6 years), HBV genotype(B vs C), grade of inflammation(G0–1 vs. G2–3), stage of fibrosis(S0–1 vs. S2–3), levels of ALT, HBsAg and HBV DNA, were chosen as candidate factors for the univariate analysis. Variables that were relatively significant(*P* < 0.5) in the univariate analysis were included in the multivariate logistic regression analysis to determine the independent factors that were associated with the treatment response (Table S3-S6). The general results of the multivariate analysis are shown in Table 3. Among the baseline variables, peg-IFN (odds ratio (OR) 4.468, confidence interval (CI) 1.135–17.583, *P* < 0.05), higher ALT levels (OR 1.268, CI 1.053–1.527, *P* < 0.05), lower HBsAg levels (OR 0.220, CI 0.068–0.715, *P* < 0.05) and younger age(< 6 years) (OR 6.177, CI 1.539–24.786, *P* < 0.05) were considered favourable factors for HBeAg SR in multivariate analysis. Similarly, peg-IFN (OR 2953.434, CI 1.302–6,702,066.994, *P* < 0.05), lower HBsAg levels (OR 0.008, CI 0.000–0.506, *P* < 0.05) and younger age(< 6 years) (OR 659.807, CI 1.371–317,523.678, P < 0.05) were considered favourable factors for HBsAg SR in multivariate analysis. As for both virological response and biological response, ETV remained as the only independent favorable factor in multivariate analysis (OR 0.222, CI 0.052–0.956, *P* < 0.05; OR 0.128, CI 0.027–0.614, *P* < 0.05 respectively).

**Table 3:** Independent factors that were associated with treatment response at week 48(non-response vs response) in CHB children using multivariate logistic regression analysis

Items	Variables	OR(95%CI)	B	P
HBeAg serological response	Initial drug (IFN vs ETV)	4.468(1.135, 17.583)	1.497	**0.032**
	ALT/ULN	1.268(1.053, 1.527)	0.237	**0.012**
	baseline log_10_HBsAg	0.220(0.068, 0.715)	−1.514	**0.012**
	Age(<6y vs ≥6y)	6.177(1.539, 24.786)	1.821	**0.010**
Virological response	Initial drug (IFN vs ETV)	0.222(0.052, 0.956)	−1.506	**0.043**
Biochemical response	Initial drug (IFN vs ETV)	0.128(0.027, 0.614)	−2.057	**0.010**
HBsAg serological response	Initial drug (IFN vs ETV)	2953.434(1.302, 6,702,066.994)	7.991	**0.043**
	baseline log_10_HBsAg	0.008(0.000, 0.506)	−4.816	**0.022**
	Age(<6y vs ≥6y)	659.807(1.371, 317,523.678)	6.492	**0.039**

*OR* Odds ratio, *CI* Confidence interval, *ETV* Entecavir, *PegIFN* Pegylated interferon, *ALT* Alanine aminotransferase, *AST* Aspartate aminotransferase, *ULN* Upper limit of normal, *HBV* Hepatitis B virus, *HBsAg* Hepatitis B surface antigen, *HBeAg* Hepatitis B envelop antigen

### Evaluation of safety

The adverse effects were recorded during the treatment course and are listed in Table S7. No patient experienced serious adverse effects that required discontinuing or changing the treatment regimen during the 48-week follow-up. Neutropenia (13/26, 50%), abnormalities in thyroid function parameters (13/26, 50%) and ALT flares (11/26, 42.31%) were the most common adverse effects in the peg-IFN group, but all were transient without subjective symptoms. Other adverse effects in patients receiving peg-IFN included pyrexia (9/26, 34.62%), elevation of autoantibody (9/26, 34.62%), fatigue, arthralgia, hair loss and rash. In the ETV group, an abnormal electrocardiogram (4 had a wandering pacemaker; 2 had left ventricle high tension) or elevation of creatine kinase isoenzymes were observed in 12 patients (12/44, 27.27%), none of whom required special treatment after evaluation by cardiologists. Neutropenia (5/44, 11.36%), ALT flares (4/44, 9.09%) and elevation of autoantibody (1/44, 2.27%) were not common in patients receiving ETV. Virological breakthrough was observed in 4 patients receiving peg-IFN (4/26, 15.38%) and 9 patients receiving ETV (9/44, 20.45%) during the treatment period.

### Elevated pretreatment IL-18 levels were associated with HBeAg SR

**Fig. 2:**
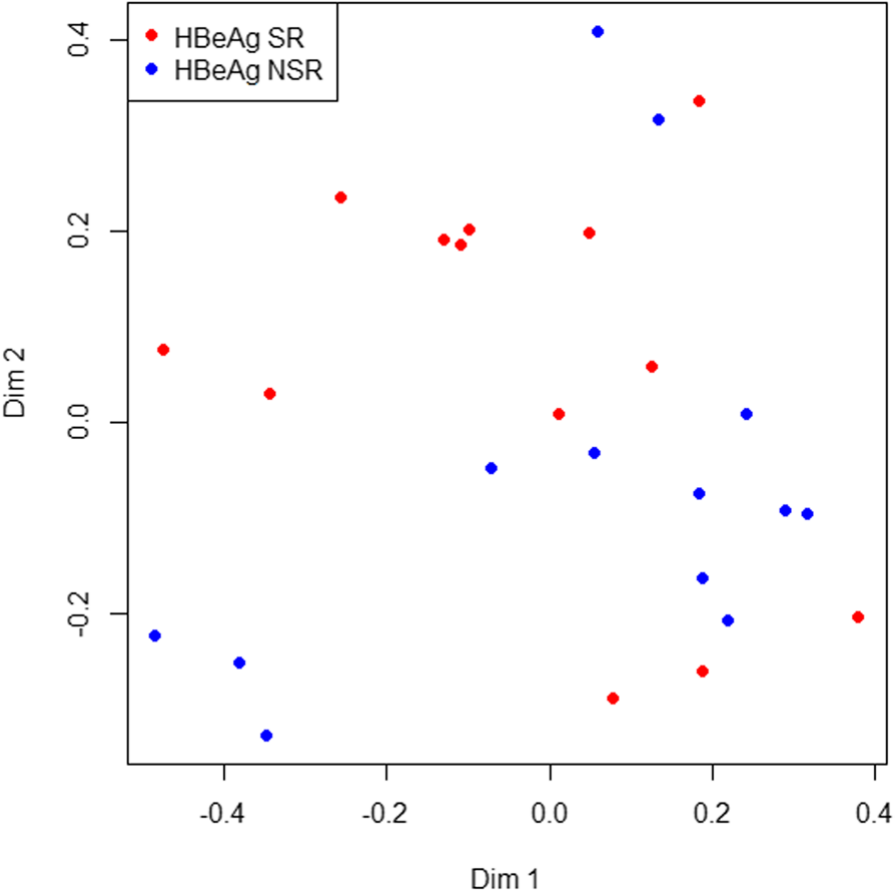
Multidimensional scaling plot displayed that serum cytokine profiles of patients with HBeAg SR (red dots) and NSR (blue dots) differed. The plot represents the proximity matrices of the random forest model and the two axes represent the first and second metric multidimensional scaling axes. SR: serological response; NSR: without serological response

**Fig. 3:**
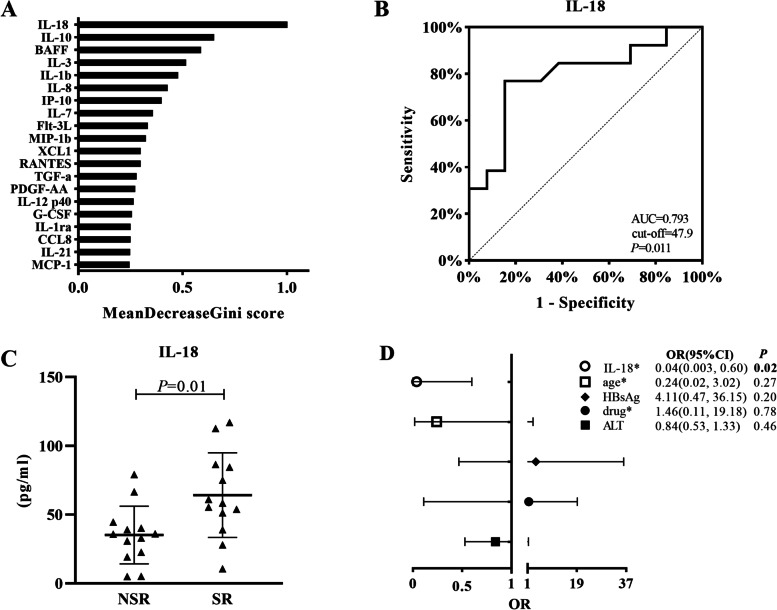
Elevated pre-treatment IL-18 level was associated with HBeAg SR. The relative importance of the top 20 important cytokines in the overall classification. The vertical axes represent the arrangement of importance among the cytokines according to the Gini scores. The horizontal axes represent the average decrease in classification accuracy as the Gini scores. **A** The receiver operating curve of IL-18 for differentiating patients with HBeAg SR and NSR. **B** The comparison of serum IL-18 levels in patients with HBeAg SR and NSR. Data were analyzed by the Mann-Whitney U test. **C** The binary logistic regression analysis (HBeAg SR vs HBeAg NSR) including IL-18(≥50 pg/ml vs < 50 pg/ml), age(<6y vs ≥ 6y), drug (pegylated interferon vs entecavir), HBsAg (high vs low) and ALT (high vs low). SR:serological response; NSR:without serological response; AUC: area under the curve; OR: odds ratio; CI: confidence interval; ALT: alanine aminotransferase; HBsAg: hepatitis B surface antigen

The classification between patients who achieved HBeAg SR(*n* = 13) and those who had HBeAg NSR (*n* = 13) was conducted using a fivefold cross-validation for feature selection by random forest. The serum cytokine profiles between patients with HBeAg SR and NSR differed substantially, as shown by ROC analysis (AUC = 0.63) and multidimensional scaling plots (Fig. 2). The importance of cytokines in the overall classification was ranked by Gini score. The top 20 cytokines are shown in Fig. 3A, and IL-18, IL-10, BAFF, IL-3 and IL-1b were identified as required for maximum classification accuracy with the top 5 Gini scores. The levels of the 5 cytokines were significantly different between patients with HBeAg SR and NSR(*P* < 0.05) (Fig. S2). Among the 5 cytokines, ROC analysis of each of them revealed IL-18 as the only significant cytokine differentiating patients with HBeAg SR and NSR (AUC = 0.793, cut-off = 47.9, *P* < 0.05) (Fig. 3B). IL-18 levels were significantly higher in patients with HBeAg SR than in those with NSR (*P* < 0.05) (Fig. 3C). In the multivariate logistic regression model that included the former independent factors for HBeAg SR (age, drug, levels of ALT and HBsAg) and IL-18, higher levels of IL-18 (≥50 pg/ml) remained the only significant factor associated with HBeAg SR (OR 0.04, CI 0.003–0.60, *P* < 0.05) (Fig. 3D).

## Discussion

We are the first to conduct a comparison study on the efficacy and safety of peg-IFN and ETV therapy over a period of 48 weeks in children and adolescents with CHB. In regard to treatment response, ETV was the only independent favourable factor for both virological and biochemical response, which suggested its potent efficacy in inhibiting HBV DNA replication and improving liver inflammation. However, SR especially HBsAg SR, which is an important marker for a functional cure, was more frequently achieved in patients receiving peg-IFN than in those receiving ETV, which was in accordance with former studies in adults [8, 14]. HBeAg or HBsAg SR are important treatment endpoints, as they are usually indications of immune control over HBV and increased survival in the long term. Previous studies have reported that HBeAg seroconversion rates could reach 25–36.3% and HBsAg seroconversion rates could reach 2.3–6% in patients receiving peg-IFN, while seroconversion rates were low (approximately 20% and 0–2% for HBeAg and HBsAg SR, respectively) and less durable in patients receiving ETV [6, 15, 16]. Currently, few studies have performed head-to-head comparisons between the 2 drugs in CHB patients. In this retrospective comparison study in CHB children, we found that peg-IFN was associated with a better HBeAg or HBsAg SR than ETV in the multivariate analysis. The underlying mechanism of the better SR of peg-IFN may be due to its antiviral activity against intracellular HBV covalently closed circular DNA (cccDNA) [17, 18]. Recent studies have revealed that IFN can not only induce cccDNA degradation by the concerted action of APOBEC-mediated deamination and ISG20-mediated nuclease digestion [19], but also repress cccDNA transcription by epigenetic modification such as by modulating GCN5-mediated succinylation of histones in cccDNA minichromosomes [20, 21]. However, HBV has also evolved an immune escape mechanism and can generate multiple factors, such as viral proteins, to disrupt IFN signalling pathways. The stable minichromosome cccDNA in the hepatocyte nucleus and the unique replication strategy of HBV lead to the generation of large amounts of HBV viral proteins, including HBsAg. High and persistent HBsAg levels in the circulating blood can impair the host HBV-specific immune response and contribute to the immune tolerance and chronicity of HBV infection [22], while reduction of HBsAg could help recover the host anti-HBV immune response and predict the treatment response in the early phase [23]. In our study, lower HBsAg levels at baseline were positively associated with both HBeAg and HBsAg SR, which similarly indicated the value of quantitative HBsAg in guiding antiviral therapy in CHB children and adolescents. Moreover, younger age(< 6 years) was also a favourable factor for both HBeAg and HBsAg SR. Studies have reported that children and adolescents, especially younger children, seemed to have higher rates of SR than adults [24–26], which is possibly due to the relatively short incubation time of HBV and the less exhausted HBV-specific immune response of the host [27, 28]. A higher level of ALT was an independent favourable factor for HBeAg SR, which was also in line with previous studies [29, 30]. Subgroup analysis exploring the relationship between different ALT levels and HBeAg SR could be conducted in future studies with larger samples.

Even though children and adolescents have a relatively good probability of achieving the HBeAg and HBsAg SR, the total rates of response are still lower than desired. To optimize the current therapy, selecting potential responders before treatment and adapting the regimen during the early phase of treatment are necessary. Viral markers such as HBV genotype, quantitative HBV DNA, HBeAg and HBsAg have been widely explored to predict the treatment response, and the value of host immune factors in the prediction has also aroused attention recently. Previous studies have reported that the differentially expressed genes in liver tissues that associated with treatment response were mostly enriched in the cytokine-mediated immune response pathway [31]. Impaired HBV-specific immune responses of NK cells and T cells and poor antiviral cytokine production contribute to the chronicity of HBV infection and might influence the treatment response [28, 32]. To evaluate the association between host immune profiles and different clinical outcomes, we measured a panel of serum cytokines in 26 patients before treatment. We found that pretreatment cytokine profiles could significantly differentiate HBeAg SR from HBeAg NSR by a random forest model. Among the measured cytokines, IL-18 was the most important in the classification with the highest Gini scores and it remained significant in the ROC analysis. After adjusting for other important virological and clinical factors, the elevated serum level of IL-18 at baseline was still independently associated with HBeAg SR. IL-18, a proinflammatory cytokine, can stimulate IFN-γ release by NK cells, T cells, dendritic cells and B cells with inflammasome activation. Previous studies have proposed that IL-18 may have a close interaction with HBeAg and may be valuable in predicting HBeAg SR in anti-HBV therapy. It has been reported that HBeAg can suppress IL-18-mediated IFN-γ expression in NK cells via modulation of the NF-κB signalling pathway, which facilitates the persistent infection of HBV [33]. In turn, elevated IL-18 can inhibit HBV replication in murine and hepatoma cell lines through induction of IFN-γ and other unrevealed mechanisms [34, 35]. Clinical studies have also proven that the level of IL-18 was significantly elevated before HBeAg SR in HBV monoinfected and HBV-HIV coinfected patients receiving antiviral therapy [36, 37]. Evaluating serum cytokines such as IL-18 may be a complementary tool to virological and clinical parameters and help identify potential responders and the optimal timing of anti-HBV treatment.

No serious adverse effects of the two drugs were observed in this retrospective study. It seemed that patients receiving peg-IFN had more frequent and various adverse effects in the early period of treatment. ALT flares tended to be more frequently observed in peg-IFN group than in the ETV group (11/26 vs. 4/44) and mostly occurred in the early phase of treatment ranging from week 3 to week 23(week 8.1 ± 1.3). Among the patients who had ALT flares during treatment, higher rates of HBeAg SR were observed in the peg-IFN group than in the ETV group. That is, 6 out of 11 patients in the peg-IFN group and 1 out of 4 in the ETV group achieved HBeAg SR, all of which occurred close to the time of the ALT flares, ranging from week 5 to week 27 (week 15.4 ± 3.1). This finding was in line with a recent observational study of ALT flares in adults with CHB, which detected that ALT flares during peg-IFN treatment were associated with the decline of HBsAg and HBV RNA and could predict subsequent HBsAg loss [38]. Regarding virological breakthrough, it is worth noting that the occurrence of virological breakthrough was relatively frequent in the ETV group, mostly due to poor patient compliance. It is possible that patients, especially children receiving ETV, easily forget to take the drug, as it is taken daily. Therefore, patient education and practice should be further strengthened for the guardians of paediatric patients, especially for those who take ETV therapy, to improve the long-term prognosis.

Despite the positive results, there are a few limitations of this clinical study. First, the follow-up and efficacy evaluation of the 2 drugs were performed within 48 weeks and the outcomes were unclear for the long term. However, it might be reasonable to set the endpoints of a comparison study at a time when both drugs are not discontinued, because the recommended treatment courses of the 2 drugs are currently quite different. Peg-IFN is usually given as a finite course of 1 year, while ETV is usually given as a continuous course of at least 2 years [16]. It remains controversial whether a more prolonged peg-IFN course might be more effective. Recent studies have reported that a prolonged treatment course (72–96 weeks) of peg-IFN had higher rates of HBsAg SR and sustained virological response in HBeAg-negative adults with genotype D or E [39, 40]. In addition, although we detected that there were significant relationships between pretreatment cytokine profiles and treatment response, it is worth noting that host immune profiles were altered in a complex and dynamic manner. Monitoring the longitudinal changes in host immune profiles might be valuable for understanding how the host immune system reacts to antiviral therapies. Future prospective studies with larger sample sizes and longer follow-ups should be conducted.

In summary, we found that initial peg-IFN therapy was more effective in achieving HBsAg and HBeAg SR but not virological and biochemical responses compared to ETV during a 48-week follow-up in children and adolescents with CHB. These results further highlighted the role of IFN or peg-IFN in achieving a functional cure of CHB. We also found that significantly different cytokine profiles and IL-18 levels between patients with HBeAg SR and NSR. Individual variability in immune status and response to antiviral therapy suggests that more investigations are required to identify potential responders before treatment. Immunological markers such as elevated pretreatment IL-18 might supplement the viral markers and serve as promising predictors of the CHB treatment response.

## Supplementary Information

**Additional file 1: Fig. S1.** Rates of patients with treatment response (HBeAg serological response, HBsAg serological response, virological response and biochemical response) at baseline, week 4, 12, 24 and 48 in the pegylated interferon and entecavir treatment group, respectively. HBeAg: hepatitis B e antigen; HBsAg: hepatitis B surface antigen.

**Additional file 2: Fig. S2.** The levels of differentially expressed cytokines between patients with HBeAg serological response (SR) and without HBeAg serological response (NSR).

**Additional file 3: Table S1.** List of cytokines measured by quantibody® array kit. **Table S2.** Median levels of serum HBV DNA and ALT at each visit (weeks 4, 12, 24 and 48). **Table S3.** Univariate and multivariate analysis for HBeAg serological response at week 48(non-response vs response) in CHB children. **Table S4.** Univariate and multivariate analysis for virological response at week 48(non-response vs response) in CHB children. **Table S5.** Univariate and multivariate analysis for biochemical response at week 48(non-response vs response) in CHB children. **Table S6.** Univariate and multivariate analysis for HBsAg serological response at week 48(non-response vs response) in CHB children. **Table S7.** Cumulative adverse events in peg-IFN and ETV groups.

## Data Availability

Datasets used and analyzed during the current study are available from the corresponding authors on reasonable request.
