# Supplementary figures and images for: The efficacy of pegylated interferon alpha-2a and entecavir in HBeAg-positive children and adolescents with chronic hepatitis B
Source: BMC Pediatr. 2022 Jul 20;22:426. doi: 10.1186/s12887-022-03482-0 (PMC9297582; doi:10.1186/s12887-022-03482-0)

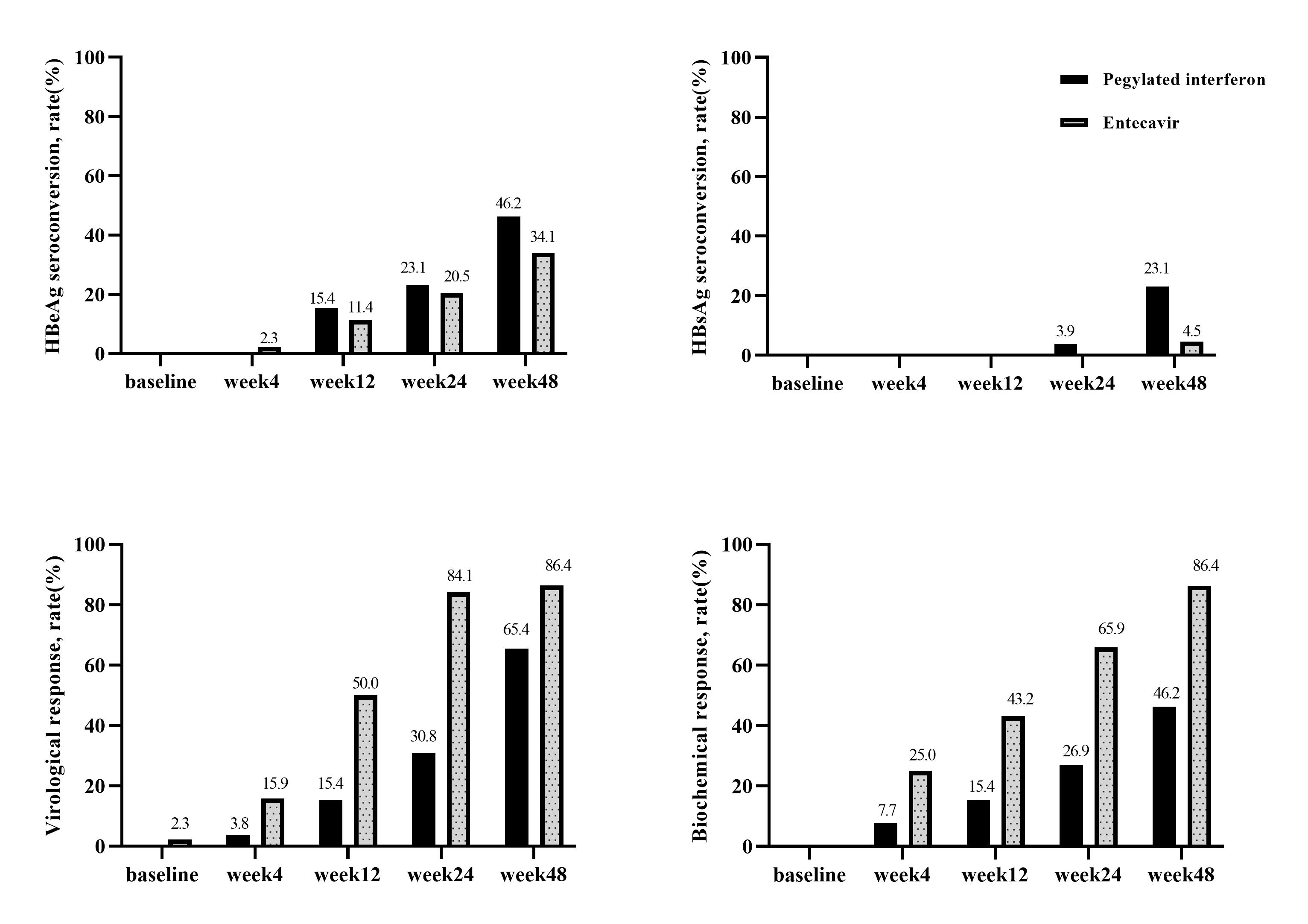

Supplement: Supplementary file 1 — Additional file 1: Fig. S1. Rates of patients with treatment response (HBeAg serological response, HBsAg serological response, virological response and biochemical response) at baseline, week 4, 12, 24 and 48 in the pegylated interferon and entecavir treatment group, respectively. HBeAg: hepatitis B e antigen; HBsAg: hepatitis B surface antigen. [file 12887_2022_3482_MOESM1_ESM.jpg]

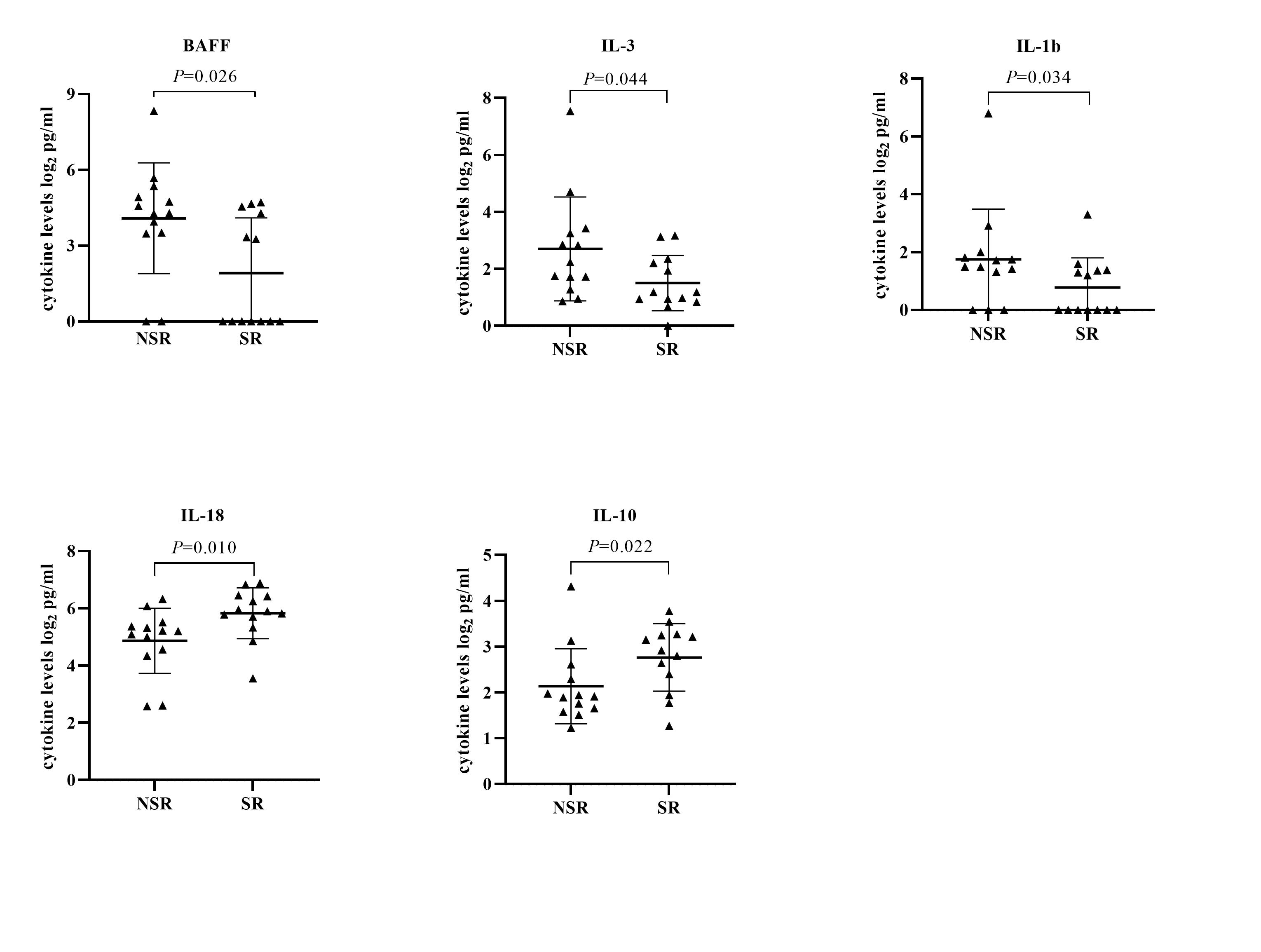

Supplement: Supplementary file 2 — Additional file 2: Fig. S2. The levels of differentially expressed cytokines between patients with HBeAg serological response (SR) and without HBeAg serological response (NSR). [file 12887_2022_3482_MOESM2_ESM.jpg]
