# Supplementary material for: The efficacy of pegylated interferon alpha-2a and entecavir in HBeAg-positive children and adolescents with chronic hepatitis B
Source: BMC Pediatr. 2022 Jul 20;22:426. doi: 10.1186/s12887-022-03482-0 (PMC9297582; doi:10.1186/s12887-022-03482-0)
Supplement: Supplementary file 3 — Additional file 3: Table S1. List of cytokines measured by quantibody® array kit. Table S2. Median levels of serum HBV DNA and ALT at each visit (weeks 4, 12, 24 and 48). Table S3. Univariate and multivariate analysis for HBeAg serological response at week 48(non-response vs response) in CHB children. Table S4. Univariate and multivariate analysis for virological response at week 48(non-response vs response) in CHB children. Table S5. Univariate and multivariate analysis for biochemical response at week 48(non-response vs response) in CHB children. Table S6. Univariate and multivariate analysis for HBsAg serological response at week 48(non-response vs response) in CHB children. Table S7. Cumulative adverse events in peg-IFN and ETV groups. [file 12887_2022_3482_MOESM3_ESM.docx]

**Table S1. List of cytokines measured by quantibody® array kit**

| Numbers |  | Numbers |  |
| --- | --- | --- | --- |
| 1 | IP-10（CXCL10） | 27 | GRO |
| 2 | MDC (CCL22) | 28 | IFN-α2 |
| 3 | TNF-α | 29 | IFN-γ |
| 4 | TNF-β | 30 | MCP-1 |
| 5 | IL-1α | 31 | MCP-3 |
| 6 | IL-1β | 32 | MIP-1α（CCL3） |
| 7 | IL-1ra | 33 | MIP-1β（CCL4） |
| 8 | IL-2 | 34 | PDGF-AA |
| 9 | IL-3 | 35 | PDGF-AB |
| 10 | IL-4 | 36 | PDGF-BB |
| 11 | IL-5 | 37 | RANTES（CCL5） |
| 12 | IL-6 | 38 | TGF-α |
| 13 | IL-7 | 39 | VEGF |
| 14 | IL-8 | 40 | EGF |
| 15 | IL-9 | 41 | Eotaxin |
| 16 | IL-10 | 42 | Flt-3 ligand |
| 17 | IL-12 (p40) | 43 | Fractalkine（CX3CL1） |
| 18 | IL-12 (p70) | 44 | BAFF |
| 19 | IL-13 | 45 | APRIL |
| 20 | IL-15 | 46 | CCL8 |
| 21 | IL-17 | 47 | CD27L |
| 22 | IL-18 | 48 | IGF-I |
| 23 | IL-21 | 49 | XCL1 |
| 24 | IL-32α | 50 | CXCL9 |
| 25 | G-CSF | 51 | CXCL13 |
| 26 | GM-CSF |  |  |

**Table S2. Median levels of serum HBV DNA and ALT at each visit (weeks 4, 12, 24 and 48)**

| Parameter | Peg-IFN(n=26) | | | ETV(n=44) | | |
| --- | --- | --- | --- | --- | --- | --- |
|  | HBV DNA(log10 IU/ml) | ALT/ULN | HBeAg(S/CO) | HBV DNA(log10 IU/ml) | ALT/ULN | HBeAg(S/CO) |
| Week 4 | 6.98(2.35) | 3.69(3.81) | 564.59(1273.10) | 4.32(1.65) | 2.32(2.88) | 667.49(1137.57) |
| Week 12 | 5.39(3.04) | 2.29(2.53) | 288.71(943.17) | 3.24(1.47) | 1.19(1.44) | 307.98(845.71) |
| Week 24 | 4.11(3.90) | 1.64(1.29) | 162.23(872.60) | 2.60(0.56) | 0.80(0.61) | 33.75(323.28) |
| Week 48 | 2.60(1.18) | 1.29(1.57) | 4.85(139.30) | 2.60(0.50) | 0.67(0.39) | 4.25(224.11) |

Continuous data were expressed as median (inter-quartile range). ETV: entecavir; Peg-IFN: pegylated interferon; ALT: alanine aminotransferase; ULN:upper limit of normal; HBV: hepatitis B virus; HBeAg, hepatitis B envelop antigen.

**Table S3. Univariate and multivariate analysis for HBeAg serological response at week 48(non-response vs response) in CHB children**

| Variables | Univariate analysis | | *P* | Multivariate analysis | | *P* |
| --- | --- | --- | --- | --- | --- | --- |
|  | OR(95%CI) | B |  | OR(95%CI) | B |  |
| Initial drug(IFN vs ETV) | 1.657(0.615, 4.466) | 0.505 | **0.318** | 4.468(1.135, 17.583) | 1.497 | **0.032** |
| ALT/ULN | 1.196(1.107, 1.406) | 0.179 | **0.030** | 1.268(1.053, 1.527) | 0.237 | **0.012** |
| baseline log_10_HBsAg | 0.238(0.093, 0.614) | -1.434 | **0.003** | 0.220(0.068, 0.715) | -1.514 | **0.012** |
| baseline log_10_HBV DNA | 0.598(0.360, 0.993) | -0.515 | **0.047** |  |  |  |
| Age(<6y vs ≥6y) | 2.333(0.872,6.246) | 0.847 | **0.092** | 6.177(1.539, 24.786) | 1.821 | **0.010** |
| Genotype(B vs C) | 2.250(0.604, 8.379) | 0.811 | 0.527 |  |  |  |
| Gender（male vs female) | 0.862(0.322, 2.310) | -0.149 | 0.768 |  |  |  |
| G0-1 vs G2-3 | 0.720(0.272, 1.906) | -0.328 | 0.509 |  |  |  |
| S0-1 vs S2-3 | 0.370(0.134, 1.026) | -0.994 | **0.056** |  |  |  |

**Table S4. Univariate and multivariate analysis for virological response at week 48(non-response vs response) in CHB children**

| Variables | Univariate analysis | | *P* | Multivariate analysis | | *P* |
| --- | --- | --- | --- | --- | --- | --- |
|  | OR(95%CI) | B |  | OR(95%CI) | B |  |
| Initial drug(IFN vs ETV) | 0.298(0.092, 0.971) | -1.210 | **0.045** | 0.222(0.052, 0.956) | -1.506 | **0.043** |
| ALT/ULN | 1.425(1.042, 1.950) | 0.354 | **0.027** |  |  |  |
| baseline log_10_HBsAg | 0.184(0.050, 0.672) | -1.875 | **0.010** |  |  |  |
| baseline log_10_HBV DNA | 0.405(0.174, 0.940) | -0.905 | **0.035** |  |  |  |
| Age(<6y vs ≥6y) | 1.161(0.363, 3.713) | 0.150 | 0.801 |  |  |  |
| Gender（male vs female) | 1.079(0.336, 3.469) | 0.076 | 0.898 |  |  |  |
| Genotype(B vs C) | 0.301(0.057, 1.574) | -1.202 | 0.515 |  |  |  |
| G0-1 vs G2-3 | 0.677(0.215, 2.131) | -0.389 | 0.505 |  |  |  |
| S0-1 vs S2-3 | 0.231(0.047, 1.124) | -1.466 | **0.070** |  |  |  |

**Table S5. Univariate and multivariate analysis for biochemical response at week 48(non-response vs response) in CHB children**

| Variables | Univariate analysis | | *P* | Multivariate analysis | | *P* |
| --- | --- | --- | --- | --- | --- | --- |
|  | OR(95%CI) | B |  | OR(95%CI) | B |  |
| Initial drug(IFN vs ETV) | 0.135(0.043, 0.430) | -2.000 | **0.001** | 0.128(0.027, 0.614) | -2.057 | **0.010** |
| ALT/ULN | 1.113(0.928, 1.335) | 0.107 | **0.249** |  |  |  |
| baseline log_10_HBsAg | 1.167(0.571, 2.382) | 0.325 | 0.672 |  |  |  |
| baseline log_10_HBV DNA | 0.807(0.471, 1.382) | -0.214 | **0.435** |  |  |  |
| Age(<6y vs ≥6y) | 1.179(0.411, 3.383) | 0.164 | 0.760 |  |  |  |
| Gender（male vs female) | 1.455(0.507, 4.170) | 0.375 | **0.486** |  |  |  |
| Genotype(B vs C) | 0.479(0.112, 2.051) | -0.736 | **0.321** |  |  |  |
| G0-1 vs G2-3 | 0.785(0.277, 2.226) | -0.242 | 0.649 |  |  |  |
| S0-1 vs S2-3 | 1.418(0.483, 4.156) | 0.349 | 0.525 |  |  |  |

**Table S6. Univariate and multivariate analysis for HBsAg serological response at week 48(non-response vs response) in CHB children**

| Variables | Univariate analysis | | *P* | Multivariate analysis | | *P* |
| --- | --- | --- | --- | --- | --- | --- |
|  | OR(95%CI) | B |  | OR(95%CI) | B |  |
| Initial drug(IFN vs ETV) | 6.300(1.166, 34.026) | 1.841 | **0.032** | 2953.434(1.302, 6702066.994) | 7.991 | **0.043** |
| ALT/ULN | 0.656(0.409, 1.054) | -0.421 | **0.081** |  |  |  |
| baseline log_10_HBsAg | 0.283(0.108, 0.743) | -1.261 | **0.010** | 0.008(0.000, 0.506) | -4.816 | **0.022** |
| baseline log_10_HBV DNA | 0.613(0.341, 1.102) | -0.490 | **0.102** |  |  |  |
| Age(<6y vs ≥6y) | 4.750(0.885, 25.484) | 1.558 | **0.069** | 659.807(1.371, 317523.678) | 6.492 | **0.039** |
| Gender（male vs female) | 1.053(0.230, 4.812) | 0.051 | 0.947 |  |  |  |
| Genotype(B vs C) | 107698324.0(0, /) | 18.495 | 0.998 |  |  |  |
| G0-1 vs G2-3 | 2.160(0.474, 9.847) | 0.770 | **0.320** |  |  |  |
| S0-1 vs S2-3 | 0.854(0.186, 3.922) | -0.158 | 0.839 |  |  |  |

**Footnote of Table S3-S6.**

Variables that were relatively significant in the univariate analysis(with *P*-values <0.5) were marked in bold and included in the multivariate logistic regression analysis.

G: grade of inflammation; S:stage of fibrosis in liver histo logy were evaluated according to Scheuer’s criteria. OR: odds ratio; CI:confidence interval; ETV: entecavir; Peg-IFN:pegylated interferon; ALT: alanine aminotransferase; AST:aspartate aminotransferase; ULN:upper limit of normal; HBV: hepatitis B virus; HBsAg, hepatitis B surface antigen; HBeAg, hepatitis B envelop antigen.

**Table S7. Cumulative adverse events in peg-IFN and ETV groups**

| Adverse events, n(%) | peg-IFN(n=26) | ETV(n=44) |
| --- | --- | --- |
| ALT flares(>5ULN) | 11(42.31%) | 4(9.09%) |
| Thyroid dysfunction | 13(50.00%) | / |
| Neutropenia(<1.5) | 13(50.00%) | 5(11.36%) |
| Pyrexia | 9(34.62%) | / |
| Auto-antibody abnormality | 9(34.62%) | 1(2.27%) |
| Fatigue and poor appetite | 4(15.38%) | / |
| Arthralgia | 4(15.38%) | / |
| Hair loss | 3(11.54%) | / |
| Dermatologic | 3(11.54%) | / |
| Cardiac | / | 12(27.27%) |
| Virologic breakthrough | 4(15.38%) | 9(20.45%) |

ALT: alanine aminotransferase; Virologic breakthrough: HBV DNA level increase of more than 1 log10 IU/ml during therapy, usually caused by poor adherence to treatment or emergence of a drug-resistant HBV mutant.
